# Compost spatial heterogeneity promotes evolutionary diversification of a bacterium

**DOI:** 10.1111/jeb.13722

**Published:** 2020-11-18

**Authors:** Stineke van Houte, Daniel Padfield, Pedro Gómez, Adela M. Luján, Michael A. Brockhurst, Steve Paterson, Angus Buckling

**Affiliations:** ^1^ ESI and CEC, Biosciences University of Exeter Penryn UK; ^2^ Department of Animal and Plant Sciences University of Sheffield Sheffield UK; ^3^ Institute of Integrative Biology University of Liverpool Liverpool UK

**Keywords:** adaptive radiation, *Pseudomonas fluorescens*, soil, spatial heterogeneity

## Abstract

Spatial resource heterogeneity is expected to be a key driver for the evolution of diversity. However, direct empirical support for this prediction is limited to studies carried out in simplified laboratory environments. Here, we investigate how altering spatial heterogeneity of potting compost—by the addition of water and mixing—affects the evolutionary diversification of a bacterial species, *Pseudomonas fluorescens*, that is naturally found in the environment. There was a greater propensity of resource specialists to evolve in the unmanipulated compost, while more generalist phenotypes dominated the compost–water mix. Genomic data were consistent with these phenotypic findings. Competition experiments strongly suggest these results are due to diversifying selection as a result of resource heterogeneity, as opposed to other covariables. Overall, our findings corroborate theoretical and in vitro findings, but in semi‐natural, more realistic conditions.

## 1 INTRODUCTION

Theoretical work suggests that spatial heterogeneity in resources can lead to the evolution and maintenance of genotypic diversity through diversifying selection (Amarasekare, 2003; Chesson, 2000a; Chesson, 2000b; Chesson, 2018; Kassen, 2002; Leimar, 2005; Smith & Hoekstra, 1980). Assuming trade‐offs in their ability to grow on spatially distributed resources, genotypes are likely to be more closely associated with the resource to which they are best adapted, resulting in greater intra‐versus inter‐genotype competition. These effects can be further amplified by a positive covariance between a genotype's maximum growth rate and its local density (Chesson, 2000a; Leimar, 2005). Spatial structure of populations can also result in diversifying selection in the absence of resource heterogeneity, assuming life history trade‐offs (e.g. colonization versus competitive abilities) and that patches show asynchronous variation in competition through time (Hastings, 1980). Evolved diversity may also be greater in spatially structured populations not as a consequence of diversifying selection, but because the resultant smaller sub‐populations increase the importance of genetic drift (Wright, 1932) and the probability of different mutations arising in different sub‐populations (Orr, 2005).

Direct tests of this theory using experimentally evolving populations of microbes in vitro are supportive of a role of spatial heterogeneity in driving the evolution of genotypic diversity. For example, greater phenotypic diversity evolved in populations of *Escherichia coli* on agar plates compared to liquid media (Habets et al., 2006; Korona et al., 1994), and in populations of *Pseudomonas fluorescens* in static relative to shaken liquid media (Rainey & Travisano, 1998). However, the relevance of in vitro studies to natural populations can always be questioned. There is a need, therefore, to understand how spatial resource heterogeneity affects microbial evolutionary diversification in more ecologically relevant environments.

For many terrestrial microbes, variation in moisture level likely plays a key role in both the degree of spatial resource heterogeneity and the extent to which populations are spatially structured. Here, we evolved the soil bacterium *Pseudomonas fluorescens* in one of its natural habitats: potting compost with its spatial structure intact, or in compost that was mixed with water. This manipulation inevitably alters the environment in additional ways than just changing spatial structure, but this is also the case for the in vitro manipulations described above. Nevertheless, the resultant changes in phenotypic diversity based on substrate use, population genomic changes and population‐level fitness assays strongly suggest that soil spatial structure results in diversifying selection for bacteria specializing on different soil resources.

## 2 MATERIALS AND METHODS

### 2.1 Strains

*Pseudomonas fluorescens* strain SBW25 (Rainey & Bailey, 1996) was used throughout the study. To generate a genetically marked SBW25 strain expressing β‐galactosidase (LacZ), Tn7‐mediated transposition was carried out to insert a LacZ gene into the *P. fluorescens att*Tn7 genomic location (Choi & Schweizer, 2006).

### 2.2 Growth conditions of the evolution experiment in compost

Isogenic *Pseudomonas fluorescens* SBW25 was grown overnight at 28 °C in King's media B (KB) in an orbital shaker (180 r.p.m.) and then centrifuged for 10 minutes at 3,500 r.p.m to produce a bacterial pellet, which was resuspended in M9 salts buffer to a final concentration of 10^8^ colony forming units (CFUs)/mL. Following our previous method (Gómez et al., 2015), six round Petri dishes each containing 25 g of twice‐autoclaved compost (John Innes no. 2) were inoculated with 5 mL of the *P. fluorescens* suspension (10^8^ CFUs/mL) to give rise to the heterogeneous environment treatment. These compost microcosms were then placed in an environmental chamber at 26 °C and 80% relative humidity. For the homogeneous environment treatment, we used six 30‐mL glass vials containing 3 g of compost mixed with 9 ml sterile water (compost–water microcosm) and inoculated each vial with 5 ml of the *P. fluorescens* suspension (10^8^ CFUs/mL). compost–water microcosms were propagated at 28 °C in an orbital shaker at 180 r.p.m. One third of each culture was serially transferred to fresh two thirds of compost and compost–water approximately every six days during the 48 days experiment, equating to a minimum of 12 generations, but likely more if there is population turn‐over at equilibrium densities. The other differences in experimental conditions between treatments (soil mass, temperature) were unavoidable for logistical reasons.

### 2.3 Sample collection

After 48 days, compost samples (2 g) were collected using a sterile spatula and mixed with 10 mL sterile M9 salts buffer and glass beads, and vortexed for 1 min. The resultant sample washes from both treatments were stored at −80 °C in 20% glycerol. To analyse bacterial densities, stocks were diluted in M9 salts buffer, plated onto KB agar and incubated for 2 days at 28 °C to determine total CFUs and CFUs per gram of compost. Differences in density between treatments were tested using two linear models with log_10_ total CFUs or CFUs/g compost as the response and adaptation environment (compost–water versus. compost) as the predictor. Total CFU count is important to understand the evolutionary potential of the population, whereas CFU/g helps evaluate which populations are most productive. From each replicate experiment, a subpopulation of 10 random bacterial clones were isolated and stored at −80 °C in 20% glycerol for further analysis.

### 2.4 Phenotypic assays

To measure phenotypic diversity (and calculate different sources of variation) in either compost–water or compost, we performed catabolic profiling using Biolog GN2 microplates (Biolog). Each plate has a set of 96 wells, each containing a different carbon source, allowing bacterial growth to be measured on multiple substrates. The Biolog plate assays are essentially a measure of a clone's functional diversity. We used the 10 random clones isolated from each of the six replicate experiments of each treatment. Each of the bacterial clones was grown individually overnight in KB broth (28 °C at 180 r.p.m). Bacteria were then diluted 1000‐fold in M9 salts buffer and incubated for 2 hours at 28 °C to starve the cells. For every clone, each well of a microplate was filled up with 150 μL of culture suspension containing the starved bacteria and incubated at 28 °C for 24 hours, after which optical density was measured at 660 nm as a proxy for bacterial growth using a plate reader (Bio‐Tek Ltd). After filtering the number of substrates to keep only those where at least minimal growth occurred in every clone (minimum OD_660_ > 0.1), the catabolic profiles (the values of OD_660_ across 92 substrates) of each clone were used in downstream analyses.

The analysis of resource‐use calculates the phenotypic variation, *V*_P_, within a population into genetic variation, *V*_G_, environmental variation, *V*_E_, and genotype‐by‐environment variation, *V*_GE_. Differences in *V*_P_, *V*_G_ and *V*_GE_ between evolution environments would indicate that changes in spatial heterogeneity result in differences in resource‐use diversity. For each population (consisting of ten randomly picked clones from a microcosm), *V*_P_ was calculated as the average (by taking the mean) Euclidean distance across all pairs of clones (Hall & Colegrave, 2006), *V*_G_ as the average variance of clone performance on each substrate (Venail et al., 2008), and *V*_E_ as the variance in the average clone performance across all substrates. We were particularly interested in genotype‐by‐environment variation, as this captures the extent to which genotypes diversified into resource‐use specialists that allows us to evaluate the amount of evolved diversity in a population. More specifically, from genotype‐by‐environment interactions, responsiveness and inconsistency can be calculated (Barrett et al., 2005; Hall & Colegrave, 2006; Venail et al., 2008). Responsiveness, *R*, indicates differences in environmental variances between clones within a population:

(1) $$R=\sum \frac{{\left(\sigma_{i}‐\sigma_{j}\right)}^{2}}{2G\left(G‐1\right)}$$

where G is the number of genotypes tested within a population and $\sigma_{i}$ and $\sigma_{j}$ are the standard deviations of environmental responses of each clone tested across all substrates. A high responsiveness value would mean some clones are generalists and some clones are specialists that use a subset of the resources used by the generalists. Resource specialization is quantified by inconsistency, *I*:

(2) $$I=\sum \frac{\sigma_{i}\sigma_{j}\left(1‐\rho_{\mathrm{ij}}\right)}{G\left(G‐1\right)}$$

where $\rho_{\mathrm{ij}}$ is the Pearson's correlation of performance across substrates between each pair of clones. High inconsistency means negative correlations between clones across environments (i.e. one clone will be better on substrate A than B, and vice versa for another clone). In instances of high inconsistency and high responsiveness, clones take advantage of different resources, and some clones are specialists, and some are generalists (see Supplementary Information for workflow of how each variance component was calculated). For each variance component, differences between compost–water and compost treatments were analysed using linear models, with evolved environment (compost–water versus. compost) as a predictor compared to a model without any predictor variables.

### 2.5 Competition assays

Competition assays were performed to look for patterns of local adaptation in evolution environments to evaluate whether bacteria were better adapted to the environment they adapted in than the other environment. For each microcosm, a mix was generated in which the 10 clones used previously (see above) were pooled together in equal amounts. This mixture was then competed 50:50 with an ancestral LacZ‐marked *P. fluorescens* clone to allow us to distinguish the mix of evolved clones from the ancestral clone. Competitions were performed in either compost microcosms or in a shaken compost–water mixture for 7 days, using a starting inoculum of 10^8^ CFUs total (i.e. 5 × 10^7^ CFUs each of ancestral clone and evolved clone mix). Samples taken at 0 (T0) and 7 (T7) days were diluted in M9 salts buffer and plated on KB agar plates containing 50 μg/mL X‐gal to allow blue/white screening. For each microcosm, the numbers of white and blue colonies were used to calculate the relative fitness of each strain (e.g. evolved clone mix or LacZ ancestor): (relative fitness = [(fraction strain A at T7) * (1–(fraction strain A at T0))]/ [(fraction strain A at T0) * (1– (fraction strain A at T7)])(Ross‐Gillespie et al., 2007). This calculation is equivalent to taking the ratio of the Malthusian parameters. To look for patterns of local adaptation, we looked at changes in relative fitness with competition (compost–water versus compost) and evolution environment (compost–water versus. compost) included as potentially interacting factors. A linear mixed effects model was used (using the *R* package *lme4* (Bates et al., 2014, p. 4)), with population included as random effect to account for the same evolved clone mix being tested across competition environments. Model selection was done using likelihood ratio tests, and targeted pairwise comparisons were carried out using the *R* package “*emmeans*” (Lenth, 2018), where we looked for differences between evolved compost populations in compost–water and compost conditions, evolved compost–water populations in compost–water and compost conditions, and evolved compost–water populations in compost–water conditions versus evolved compost populations in compost conditions.

### 2.6 Sequencing

To measure genotypic diversity in clones from each of the treatments, we performed whole‐genome sequencing (WGS) on pools of the 10 bacterial clones that were isolated from each replicate (pool‐seq). In parallel, WGS was carried out on (1) a single clone from each replicate and (2) all 10 individual clones from a single replicate of each treatment. This allowed us to estimate the degree of linkage between mutations for estimating diversity. Each of the 10 bacterial clones were grown individually overnight in KB broth (28 °C at 180 rpm). Next day, the cultures were diluted in M9 salts buffer to ensure they had equal densities as measured by OD_600_. Pools of each of the 10 clones were made by mixing equal volumes of each bacterial clone. Total DNA extraction (1.2 mL per sample; 12 pooled‐clone samples and 32 single‐clone samples) was performed using the Qiagen Blood and Tissue kit following the manufacturer's instructions. An Illumina HiSeq 2000 sequencer was used to generate 100 base pair (bp) paired reads from a 500 bp insert library. Reads were trimmed for the presence of Illumina adapter sequences using *Cutadapt* (v1.2.1). The reads were further trimmed using *Sickle* (v1.2) with a minimum window quality score of 20. Reads shorter than 10 bp after trimming were removed. Trimmed reads were mapped to the *P. fluorescens* SBW25 reference with *bwa‐mem* (v0.7.12‐r1039). For the clonal level sequencing, variants were identified using *GATK Haplotyper* (v3.7) and structural variants were detected using Delly2 (v0.7.7) with a subsequent cut‐off of >= 0.95 as a proportion to identify structural variants in haploid genomes. For the pool‐seq, sites prone to sequencing or mapping errors were first identified on the clonal ancestor strain using *samtools mpileup* with parameters *‐Q0* and *‐q0* (i.e. relaxed mapping and base qualities) and then filtered from all subsequent analyses. SNPs were then detected in the pooled populations using *samtools mpileup* with parameters *‐Q20* and *‐q20* (i.e. relatively strict mapping and base qualities). Indels were identified in pooled data using *scalpel* v0.5.3 (originally designed to detect indels in tumour versus somatic samples (Narzisi et al., 2014)) by comparison of evolved with ancestral samples.

### 2.7 Sequence data analysis

First, we evaluated the ability of our pooled sequencing to correctly identify the number of genetic changes observed in the clonal sequencing (genetic changes with a proportion of >= 0.95). To do this, we created a pseudo‐pool sequencing file that was based on clonal sequencing where each of the 10 clones from a pool‐seq sample had been sequenced individually, such that 10% reads from each file were added into a separate fasta file. This pseudo‐pool data were analysed using the same pool‐seq pipeline to determine the number of mutations, which should theoretically be equal to the clonal sequencing data (when the cut‐off for proportion is >= 0.1). However, whereas 12 genetic changes (8 SNPs and 4 indels) were identified across all the clonal sequencing, for the 2 replicates for which we had sequenced every clone, at least 40 SNPs were identified. With a proportion cut‐off of 0.1, we identified SNPs identified in the clonal sequencing, but always identified many more false negatives. It is unclear whether the clonal sequencing underestimates the number of genetic changes, or whether the pool‐seq pipeline overestimates such changes. As a result, we took the conservative approach of filtering identified SNPs and indels in the pool‐seq data from all the SNPs and indels identified in the clonal sequencing.

We evaluated genetic differences between treatments by calculating several commonly used metrics: (1) the genetic distance from the reference genome, calculated as the sum of the proportion of each SNP/ indel in each population; (2) the number of unique SNPs/ indels in each population; (3) alpha diversity, calculated using a modified version of the Hardy–Weinberg equilibrium, such that $\alpha =\sum (1‐p_{i}^{2}‐q_{i}^{2})$, where *i* is the position of each SNP/ indel, *p* is the proportion of the SNP/ indel and *q* is 1–*p* (Paterson et al., 2010). This is equivalent to expected heterozygosity. Differences between these metrics were analysed using 2‐sample Kruskal–Wallis tests as the data did not conform to the assumptions of normality. To test for genetic differences between populations, we performed nonmetric multidimensional scaling on the Euclidean distance matrix of SNPs/ indels and their proportions in each population using the function “*metaMDS*” in the R package “*vegan”* (Oksanen et al., 2007). Nonmetric multidimensional scaling aims to collapse information from multiple dimensions (i.e. different populations and different SNPs/indels) into just a few, allowing for differences between samples to be visualized and interpreted. Permutational ANOVA tests were run using the “*adonis*” function, with Euclidean distance as the response term and evolution environment (compost–water or compost) as the predictor variables with 9,999 iterations. Changes in beta‐diversity were examined using the “*betadisper*” function with the same response and predictor variables in the PERMANOVA.

## 3 RESULTS

**Figure 1:**
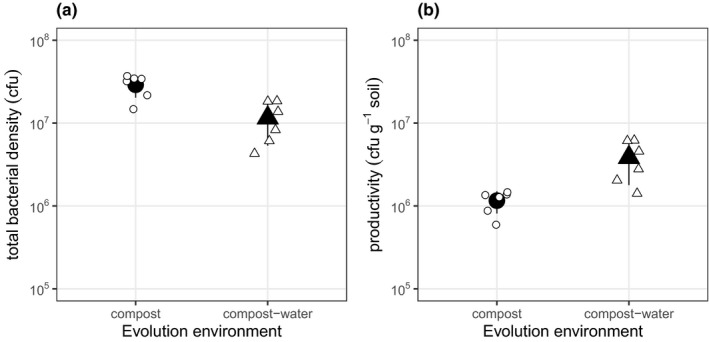
Bacterial densities of populations evolved in either compost or compost–water. (a) Total bacterial density was higher in the compost populations, but (b) density per unit compost (productivity) was higher in the compost–water populations. Points represent densities of each population (CFUs per gram of soil). Large black points are treatment means and error bars are the ± of the standard deviation. Small white points are individual population values

We evolved six replicate populations of the soil bacterium *Pseudomonas fluorescens* SBW25 in sterile potting compost (spatially heterogeneous) and a sterile compost–water mix (spatially homogeneous) for 48 days. After this period, total abundance was ~2.75 times higher in the compost populations (Figure 1a; likelihood ratio test between models with and without evolution environment as a predictor: *F*_1,10_ = 12.40, *p* = .005). However, this could be because there was more compost in these microcosms. Per gram of compost, productivity (CFU/g) was approximately 3‐fold higher in the compost–water mix (Figure 1b; likelihood ratio test between models with and without evolution environment as a predictor: *F*_1,10_ = 14.77, *p* = .003).

### 3.1 Phenotypic data

**Figure 2:**
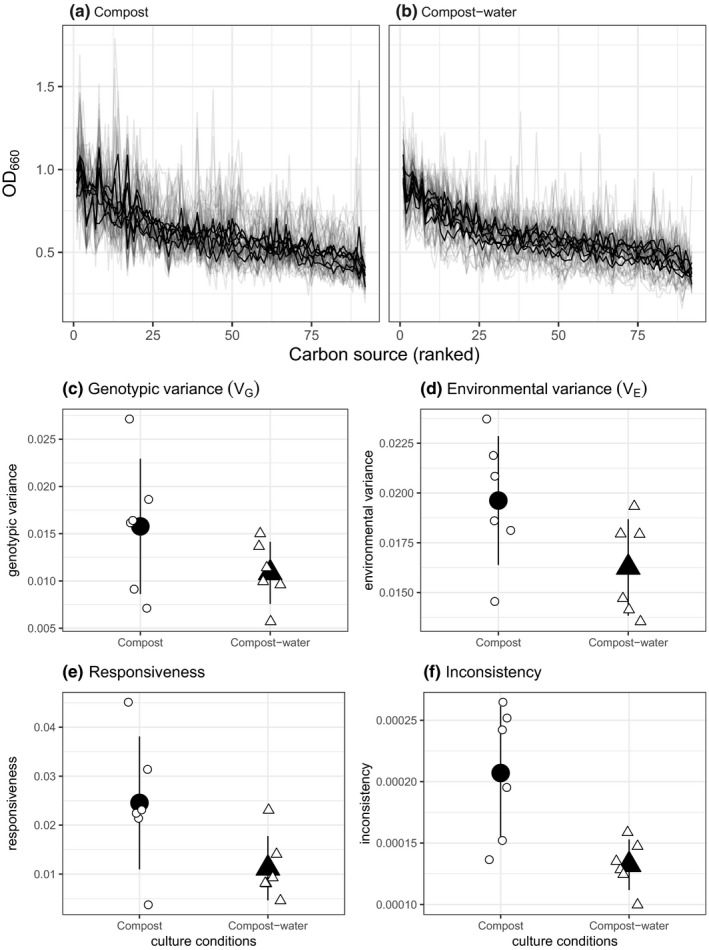
Catabolic profiles of populations evolved in either compost or compost–water. Performance of each clone evolved in either (a) compost or (b) compost–water on a variety of substrates. Total phenotypic variance was split into (c) genotypic variance (d) environmental variance and genotype × environmental components: (e) responsiveness and (f) inconsistency. Bacterial populations evolved in compost had higher inconsistency, indicating they had evolved to specialize on different resources. In (a, b), black lines represent the mean OD_660_of each population (10 clone) and grey lines represent the performance of individual clones. In (c–f) Large black points are treatment means and error bars are the ± of the standard deviation. Small white points are individual population values

To test the prediction that spatially heterogeneous (compost) environments support the evolution of greater diversification, we isolated 10 individual clones from each replicate population and measured their performance across 96 different substrates (Figure 2a,b). We then calculated total phenotypic variation (total variation in growth–final OD_660_ ‐ of all clones on all substrates) and calculated genotypic variation (*V*_G;_ the variation in mean growth on all substrates between clones), environmental variation (*V*_E;_ the variation in mean growth of all clones between substrates) and the genotype‐by‐environment interaction (*V*_GE;_ variation in growth explained by clone by substrate interaction) (Figure 2c‐f). There was no significant impact of environmental heterogeneity on total phenotypic variation (likelihood ratio test between models with and without evolution environment as a predictor: *F*_1,10_ = 2.34, *p* = .16), genotypic variation (likelihood ratio test between models with and without evolution environment as a predictor: *F*_1,10_ = 2.38, *p* = .15; Figure 2c) or environmental variation (likelihood ratio test between models with and without evolution environment as a predictor: *F*_1,10_ = 4.13, *p* = .070; Figure 2d). We further decomposed genotype‐by‐environment variation into responsiveness (the extent of variation in resource generalism versus specialism) and inconsistency (the extent of specialism). Responsiveness was not significantly impacted by environmental heterogeneity (likelihood ratio test between models with and without evolution environment as a predictor: *F*_1,10_ = 4.808, *p* = .053; Figure 2e). However, consistent with a role for spatial heterogeneity in diversification, compost environments had higher inconsistency (likelihood ratio test between models with and without evolution environment as a predictor: *F*_1,10_ = 10.026, *p* = .010; Figure 2f) compared to the compost–water environments. This suggests that the spatially heterogeneous compost environment resulted in higher diversity in resource‐use than the compost–water populations.

**Figure 3:**
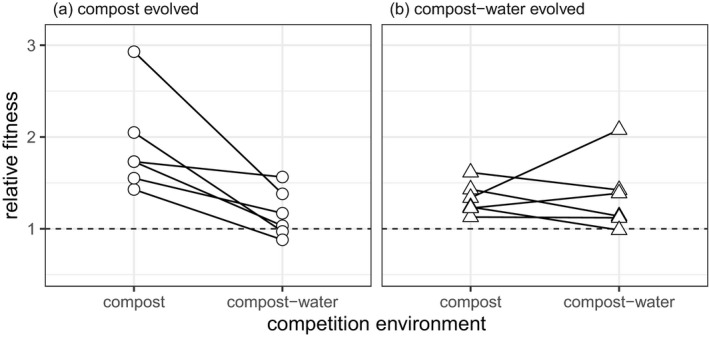
Relative fitness of populations evolved in either compost or compost–water. Points represent the relative fitness of each population. Lines show the links between each evolved population in each of its competition environments

To estimate the extent of adaptation to each environment, we competed the evolved populations against an unevolved LacZ‐marked strain in both compost and compost–water environments. Evolved populations from both treatments showed fitness gains relative to the LacZ strain (Figure 3), but there was a significant interaction between evolution and competition environments (likelihood ratio test between models with and without interaction: $\chi_{1}^{2}$=7.52, *p* = .006; Figure 3). Evolving in a compost environment increased relative fitness: compost‐evolved populations competed in compost environments (relative fitness = 1.90, 95%CI = 1.59–2.22) had a significantly higher relative fitness than compost–water‐evolved populations competed in the compost–water environment (relative fitness = 1.36, 95%CI = 1.04–1.67) (post hoc contrast between compost‐evolved population in compost environment versus. compost–water‐evolved populations in compost–water environment: t‐ratio = −2.56, *df* = 18.7, *P*_adj_ = 0.0384; Table 1). However, this greater adaptation did not transfer into the compost–water environments: compost‐evolved populations competed in compost–water environments had lower relative fitness (relative fitness = 1.17, 95%CI = 0.85–1.48) than the same populations competed in the compost environment (post hoc contrast between compost‐evolved population in compost–water versus. compost competition environment: t‐ratio = −4.02, *df* = 10, *P*_adj_ = 0.0073; Table 1). This difference was not observed in the populations evolved in compost–water conditions, with no difference in fitness between competition environments (Table 1).

**Table 1:** Results of pairwise comparisons between relative fitness of different populations that differ in evolution and competition environments (either compost or compost–water)

Contrast Evolution, competition	Estimate	SE	*df*	*t*‐ratio	*p* value
Compost, compost—compost, compost–water	0.74	0.18	10	4.02	.0073
Compost, compost—compost–water, compost–water	0.55	0.21	18.7	2.56	.0384
compost–water, compost—compost–water, compost–water	−0.03	0.18	10	−0.15	.886

Note

*p* value adjustment: Holm–Bonferroni method for 3 tests. Degrees‐of‐freedom method: Kenward–Roger

For each treatment, the evolution environment is followed by the competition environment.

### 3.2 Genomic data

**Figure 4:**
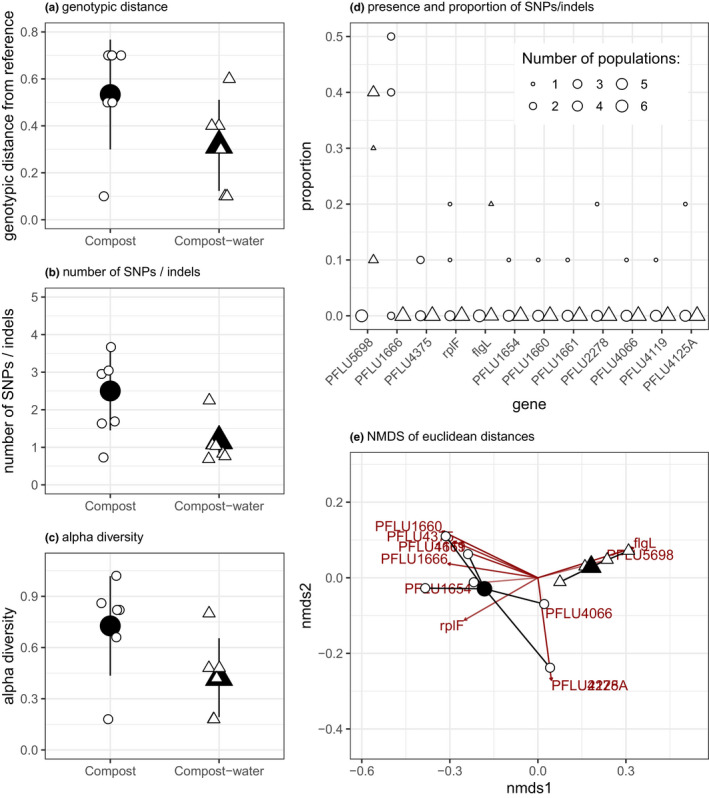
Patterns of genetic differences between populations in either compost or compost–water. The rate of evolutionary change was estimated using (a) the genetic distance from the ancestor and (b) the number of SNPs. (c) Within‐population diversity in populations evolved in compost–water and compost. (d) Distribution of SNPs and indels across all compost–water and compost populations. (e) Nonmetric multidimensional scaling (NMDS) plot of Euclidean distance between populations to visualize genomic differences between populations and treatments, with centroids (black) and populations (white). In all plots, circles represent compost populations and triangles are compost–water populations. In (a‐c) Large black points are treatment means and error bars are the ± of the standard deviation. Small white points are individual population values

Alongside differences in fitness and phenotypic diversity, we observed some genomic differences between populations evolved in compost–water and compost environments (Figure 4). In terms of genetic distance from the ancestor, compost populations had a median distance of 0.65 (IQR: 0.53–0.7), whereas compost–water populations had a median distance of 0.35 (IQR: 0.15–0.4), but this difference was not significant (Wilcoxon test: W = 6.5, *p* = .074; Figure 4a). However, there were more SNPs/ indels in the compost populations (median = 2.5, IQR = 2–3) compared to those evolved in compost–water conditions (median = 1, IQR = 1–1) (Wilcoxon test: *W* = 4.5, *p* = .029; Figure 4b). Together, this indicates that there was an increased rate of molecular evolution in the compost populations. Within‐population diversity was 0.82 (IQR = 0.81–0.85) in compost populations and 0.45 (IQR = 0.24–0.48) in compost–water populations (Wilcoxon test: W = 5.5, *p* = .052; Figure 4c). Evolution environment (compost–water versus. compost) significantly altered the genetic composition of the populations (i.e. the centroids of compost–water and compost populations are different, Figure 4e, PERMANOVA: *F*_1,10_ = 8.92, *R^2^* = 0.47, *p* = .0017). This difference was driven in large part by two genetic changes: a SNP in PFLU5698 was observed in all compost–water populations but never in the compost populations, and an indel in PFLU1666 was observed in 4 of the 6 compost populations but never in the compost–water populations (Figure 4d). There was no difference in beta‐diversity (calculated from distance‐to‐centroids between groups; Figure 4e) between compost–water and compost populations (homogeneity of multivariate dispersion ANOVA: *F*_1,10_ = 3.75, *p* = .081).

## 4 DISCUSSION

Here, we investigated how spatial heterogeneity within an ecologically relevant environment impacts diversification and adaptation of a focal bacterium (*P. fluorescens* SBW25) evolving over 48 days. Consistent with the majority of in vitro studies (Habets et al., 2006; Kassen, 2002; Rainey & Travisano, 1998) and theoretical work (Chesson, 2000b, 2018; Hedrick, 1986; Levene, 1953), we show both greater phenotypic diversity in resource‐use (Figure 2) and greater genomic diversity (Figure 4) in spatially heterogeneous potting compost compared with a more homogeneous potting compost–water mix.

Covariation of other environmental variables with spatial structure manipulations is a common problem, and we could not envisage any way to avoid this in this experimental system. For example, if we had implemented mechanical mixing of compost, this would have had a smaller confounding impact than waterlogging, but it would likely also result in a less homogeneous resource environment (Gómez et al., 2015). Another potentially confounding variable that was unavoidable here was the differences in temperature (26ºC in the compost treatment versus. 28 ºC in the compost–water treatment), but there is < 5% difference in growth of SBW25 at these two temperatures (Padfield et al., 2020). Nevertheless, we cannot unequivocally rule out that variables other than those linked to spatial structure are responsible for our results. Specifically, our manipulations resulted in higher productivity (populations reached approximately 3‐fold higher density per gram, Figure 1b) in the compost–water treatment, which can affect diversity in *P. fluorescens* (Buckling et al., 2000; Hall & Colegrave, 2006; Hall et al., 2012; Kassen et al., 2000). However, increased productivity reduces diversity through selection of dominant specialists within populations, usually characterized by greater growth of all clones overall. The patterns of resource‐use we observed are not consistent with an evolution of dominant specialists: the environmental variance in growth across resources was not increased in compost–water relative to compost, and was, if anything, lower in the compost–water treatment (Figure 2d). The results are instead consistent with selection of generalism with respect to resource‐use in the compost–water treatment, as would be predicted from reduced spatial structure (Chesson, 2000b, 2018; Hedrick, 1986; Levene, 1953).

Assuming increased diversity is a consequence of soil structure, this could be caused by both diversifying selection resulting from resource heterogeneity, and stochastic processes resulting from population sub‐division. Three lines of evidence strongly suggest the patterns of diversity are primarily a consequence of diversifying selection. First, the only significant difference in resource‐use metrics between treatments was greater within‐population specialism (“inconsistency”) within the compost compared to the compost–water treatment (Figure 2f). If stochastic processes were important, we would expect greater variance in mean growth between clones (genotypic variance) and the degree of specialization versus generalism within population (responsiveness) in the compost treatment. Second, the fitness advantage of the compost‐evolved populations in compost versus compost–water conditions, which was absent for compost–water evolved populations in compost versus compost–water conditions, suggests that specialists had a fitness advantage over generalists: a key requirement for diversifying selection but not for stochastic diversification. Finally, we have previously shown that morphologically distinct *P. fluorescens* genotypes, that differed in their resource‐use profiles, isolated from populations evolved under near‐identical conditions could re‐establish into the population from rare (Gómez & Buckling, 2013). Such negative frequency dependent fitness is a direct indication that diversity is the result of selection (Chesson, 2000a, 2018; Schluter, 2000).

We also observed that adaptation in the compost environment is greater than in the homogenous environment. This may be because total population sizes were approximately 2.75‐fold higher in compost environments which should lead to an increased mutation supply and more efficient selection, and hence a faster pace of adaptation (Fisher, 1930). Population structure can also theoretically promote adaptive evolution by allowing greater exploration of adaptive landscapes (Coyne et al., 2000; Wright, 1932) and spatial resource heterogeneity can increase the chance that beneficial mutations will encounter an environment that maximizes their fitness effect (Campos et al., 2008; Whitlock & Gomulkiewicz, 2005). On the other hand, structured populations associated with spatial heterogeneity can constrain adaptive evolution by both slowing the spread of beneficial mutations (Gordo & Campos, 2006) and increasing the role of genetic drift by reducing effective population sizes (Perfeito et al., 2008; Whitlock, 2003). In vitro experimental studies involving bacteria or viruses evolving in nutrient media provide support for increased and decreased in rates of adaptation in structured populations (Ally et al., 2014; Habets et al., 2006; Miralles et al., 1999; Perfeito et al., 2008). Given the other ways the environments differ other than spatial heterogeneity, it may simply be that mutations beneficial in the compost environment are more likely to arise.

The population genomic data are consistent with the phenotypic data. There was evidence for greater rates of molecular evolution, based on the significantly greater numbers of SNPs and indels (Figure 4), in the compost populations. There was also an indication that within‐population diversity was greater for compost versus compost–water populations, although the difference was not significant (*p* = .052). While certain genes were mutated across both treatments, different genetic changes were also selected for in the different environments, suggesting that populations evolved in compost or compost–water environments used distinct mechanisms of adaptation. A SNP in PFLU5698 was observed in all compost–water populations and resulted in an amino acid change from alanine to valine. Previous work showed that transcriptional activation of this gene, which encodes a di‐guanylate cyclase, causes a wrinkly spreader phenotype typical for mat‐forming *P. fluorescens* that colonize the air‐water interface under static growth conditions (Lind et al., 2015). Furthermore, a homolog of this gene in *Pseudomonas aeruginosa* was found to impact biofilm formation (Almblad et al., 2019). Although we never observed the formation of wrinkly spreader phenotypes during this study, these mutations may play a role in successful colonization of the air–liquid interface. The other somewhat consistent genetic change was an insertion in PFLU1666, whose predicted function is likely related to fatty acid biosynthesis, which occurred in 4 of the 6 compost populations. This indel causes a frameshift leading to a truncated protein. Transcriptional alterations of this gene were found to be associated with phenotypic switching between colony types in *P. fluorescens* (Gallie et al., 2015), and, although speculative, the mutation identified in our study may reflect a survival strategy to cope with heterogeneous environmental conditions where rapid phenotype switching is highly beneficial.

Here, we have shown that phenotypic (and to an extent, genomic) diversification is increased by spatial heterogeneity of an ecologically relevant environment, demonstrating that theoretical predictions and in vitro results can be extrapolated to semi‐natural, more realistic ecological contexts. Moreover, rates of phenotypic and molecular evolution were higher in compost environments. Our results corroborate theoretical and in vitro studies and suggest that the degree to which soil and other terrestrial environments are waterlogged may play an important role in the microevolution of microbial diversity.

### Peer Review

The peer review history for this article is available at https://publons.com/publon/10.1111/jeb.13722.

## Supporting information

Supplementary Material

## Data Availability

Raw sequencing files are archived on the European Nucelotide Archive (Experiment accession number: PRJEB41017). Final datasets and R code to recreate the figures and analyses are available on GitHub: https://bit.ly/3oVba9O and are archived on Zenodo: https://doi.org/10.5281/zenodo.4155300.
