# Supplementary material for: Compost spatial heterogeneity promotes evolutionary diversification of a bacterium
Source: J Evol Biol. 2020 Nov 18;34(2):246–55. doi: 10.1111/jeb.13722 (PMC7984246; doi:10.1111/jeb.13722)
Supplement: Supplementary file 1 — Supplementary Material [file JEB-34-246-s001.pdf]

Supplementary Information for: Compost spatial heterogeneity promotes evolutionary diversification of a bacterium

Key words: *Pseudomonas fluorescens*, soil, adaptive radiation, spatial heterogeneity

Table S1. List of substrates in the Biolog GN2 microplates.

	A	B	C	D	E	F	G	H
1	Water	i-Erythritol	D-Melibiose	Acetic Acid	p-Hydroxy-phenlyacetic Acid	Bromosuccinic Acid	L-Histidine	Urocanic Acid
2	α-Cyclodextrin	D-Fructose	β-Methyl-D-Glucoside	Cis-Aconitic Acid	Itaconic Acid	Succinamic Acid	Hydroxy-L-Proline	Inosine
3	Dextrin	L-Fucose	D-Psicose	Citric Acid	α-Ketobutyric Acid	Glucuronamide	L-Leucine	Uridine
4	Glycogen	D-Galactose	D-Raffinose	Formic Acid	α-Ketoglutaric Acid	L-Alaninamide	L-Ornithine	Thymidine
5	Tween 40	Gentiobiose	L-Rhamnose	D-Galactonic Acid Lactone	α-Ketovaleric Acid	D-Alanine	L-Phenylalanine	Phenylethyl- amine
6	Tween 80	α-D-Glucose	D-Sorbitol	D-Galacturonic Acid	D,L-Lactic Acid	L-Alanine	L-Proline	Putrescine
7	N-Acetyl-D-Galactosamine	m-Inositol	Sucrose	D-Gluconic Acid	Malonic Acid	L-Alanyl- Glycine	L-Pyroglutamic Acid	2-Aminoethanol
8	N-Acetyl-D-Glucosamine	α-D-Lactose	D-Trehalose	D- Glucosaminic Acid	Propionic Acid	L-Asparagine	D-Serine	2,3-Butanediol
9	Adonitol	Lactulose	Turanose	D-Glucuronic Acid	Quinic Acid	L-Aspartic Acid	L-Serine	Glycerol
10	L-Arabinose	Maltose	Xylitol	α- Hydroxybutyric Acid	D-Saccharic Acid	L-Glutamic Acid	L-Threonine	D,L,α-Glycerol Phosphate
11	D-Arabitol	D-Mannitol	Pyruvic Acid Methyl Ester	β- Hydroxybutyric Acid	Sebacic Acid	Glycyl-L-Aspartic Acid	D,L-Carnitine	α-D-Glucose- 1-Phosphate
12	D-Cellobiose	D-Mannose	Succinic Acid Mono-Methyl Ester	γ- Hydroxybutyric Acid	Succinic Acid	Glycyl-L-Glutamic Acid	γ-Aminobutyric Acid	D-Glucose- 6-Phosphate

Supplementary Methods: Calculating phenotypic, genotypic, environmental variance from resource-use data

2020-10-19

```
# load in packages
library(tidyverse)
library(widyr)
```

Outline Biolog plates allow us to estimate the metabolic potential of microbial communities. Typically, x number of carbon substrates are uniquely placed in each well and inoculated with a single bacterial colony. Colour change is measured and this gives a measure of bacterial growth on each substrate.

Measuring multiple clones within a population and measuring multiple populations across treatments allows us to calculate phenotypic, genetic and environmental variance. In addition, the genotype x environment interaction is especially interesting as it can represent functional diversity.

NOTE: The calculations performed here do not partition phenotypic variance into its components. The calculated metrics of V_G, V_E, responsiveness and inconsistency are unlikely to summate to V_P. However, these calculations can be used to compare relative differences in metrics between populations and treatments to gain insight into variation in resource use.

Calculations The performances of different genotypes over different environments (V_P, a measure of phenotypic diversity) and can be decomposed into genotypic (V_G), environmental (V_E) and genotype-by-environment interaction (V_{GE}).

$$V_P = V_G + V_E + V_{GE}$$

Each of these components can be calculated from the catabolic profiling of individual communities.

A catabolic profile of multiple clones in a population can be visualised using a substrate rank curve, where substrates are ranked by how well clones perform on them.

```
# have a look at the example data
glimpse(d)
#> Rows: 10
#> Columns: 11
#> $ clone        <dbl> 1, 2, 3, 4, 5, 6, 7, 8, 9, 10
#> $ substrate_1  <dbl> 0.370, 0.341, 0.304, 0.357, 0.463, 0.283, 0.352, 0.354...
#> $ substrate_2  <dbl> 0.583, 0.384, 0.418, 0.376, 0.449, 0.301, 0.618, 0.396...
#> $ substrate_3  <dbl> 0.737, 0.470, 0.440, 0.438, 0.479, 0.346, 0.418, 0.458...
#> $ substrate_4  <dbl> 0.842, 0.487, 0.512, 0.507, 0.581, 0.425, 0.405, 0.590...
#> $ substrate_5  <dbl> 0.971, 0.776, 0.699, 0.866, 0.883, 0.677, 0.836, 0.846...
#> $ substrate_6  <dbl> 0.702, 0.511, 0.444, 0.482, 0.479, 0.457, 0.486, 0.647...
#> $ substrate_7  <dbl> 0.966, 0.574, 0.572, 0.570, 0.702, 0.526, 0.608, 0.570...
#> $ substrate_8  <dbl> 0.979, 0.853, 0.889, 0.878, 0.894, 0.494, 0.851, 0.888...
#> $ substrate_9  <dbl> 0.588, 0.631, 0.318, 0.484, 0.503, 0.414, 0.468, 0.384...
#> $ substrate_10 <dbl> 0.653, 0.330, 0.315, 0.331, 0.403, 0.303, 0.433, 0.334...
```

```r
# stack data and add mean column
d_stack <- gather(d, C_source, OD, starts_with("sub")) %>% group_by(C_source) %>%
    mutate(mean = mean(OD)) %>% ungroup()

ggplot(d_stack) + geom_line(aes(as.numeric(forcats::fct_reorder(factor(C_source),
    mean, .desc = TRUE)), OD, group = clone), alpha = 0.75) +
    theme_bw(base_size = 12) + labs(x = "Substrate (ranked by mean performance)",
    y = "Performance")
```

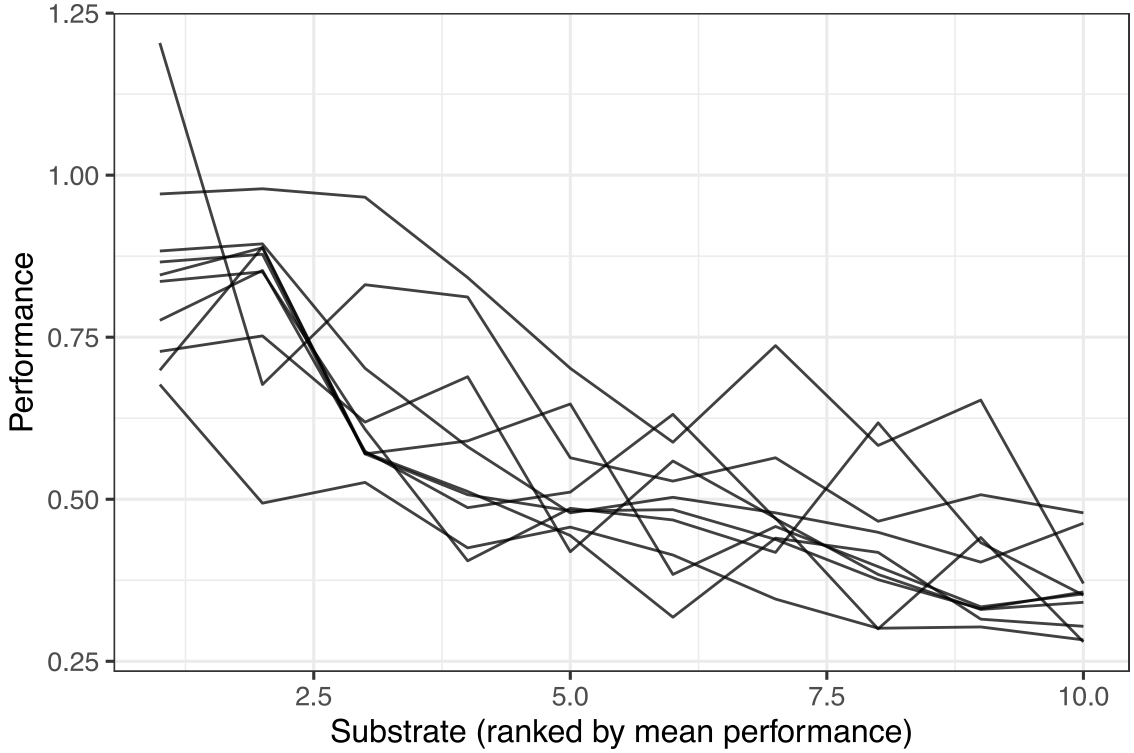

Figure 1: **Figure 1. Patterns of resource use between clones within a population.** Substrate rank performance curves for ten clones across ten substrates. Substrates are ranked by the mean value across all clones.

The slope of this curve indicates environmental variance and the width of the band of curves reflects genetic variance (Barrett, 2005). As opposed to previous versions of the curve (Barrett, 2005), this curve orders substrates in terms of their average rank, allowing us to see instances where clones have specialised on a different resource.

Changes in the rank of substrates and slope of the curve across clones within a population represent the genotype-by-environment interaction.

Calculating phenotypic diversity Phenotypic diversity, V_P, is calculated as the average Euclidean distance across all pairs of clones in a population (Hall & Colegrave, 2006). For two genotypes from the same population, the Euclidean distance is the square root of the sum of the squared differences in OD between the two genotypes across all substrates. Biologically, this measures the differences in the metabolic profiles of two genotypes.

The Euclidean method estimates the distance between two genotypes, A and B as:

$$d(A,B) = \sum_{i=1}^{j}((a_i - b_i)^2)^{1/2}$$

where a_i and b_i are the performances of clones A and B on substrate i respectively, and j is the total number of substrates.

This can be calculated easily using tools from the **tidyverse** and **widyr::pairwise_dist()**.

```
# calculate the euclidean distance of each pair of clones in
# a population
pop_dists_df <- d_stack %>% pairwise_dist(clone, C_source, OD,
    upper = FALSE) %>% rename(clone_i = item1, clone_j = item2)
#> Warning: `tbl_df()` is deprecated as of dplyr 1.0.0.
#> Please use `tibble::as_tibble()` instead.
#> This warning is displayed once every 8 hours.
#> Call `lifecycle::last_warnings()` to see where this warning was generated.

# create average phenotypic diversity per population
V_P <- mean(pop_dists_df$distance)

V_P
#> [1] 0.4925841
```

Calculating genetic variance Genetic variance V_G is calculated as the average variance of clone performance across all substrates (Venail *et al.*, 2008).

$$V_G = \frac{\sum_{i=1}^{j} Var(substrate_i)}{j}$$

Where $Var(substrate_i)$ is the variance of clone performance on substrate i and j is the total number of substrates tested.

```
# variance of OD across each genotype averaged over all the
# environments
V_G <- group_by(d_stack, C_source) %>% summarise(V_G = var(OD)) %>%
    ungroup()

# take the mean of the variances
V_G_pop <- mean(V_G$V_G)
V_G_pop
#> [1] 0.01426297
```

Calculating environmental variance Environmental variance, V_E is calculated as the variance in the average clone performance across all substrates.

```
# first calculate mean performance on each substrate
V_E <- group_by(d_stack, C_source) %>% summarise(V_E = mean(OD)) %>%
    ungroup()
```

```r
# second take the variance of this mean
V_E_pop <- var(V_E$V_E)
V_E_pop
#> [1] 0.02808666
```

Calculating genotype-by-environment interaction The genetic-by-environment interaction occurs as clones react differently to different environments. The V_{GE} interaction captures the extent to which clones diversified into resource-use specialists and allows us to evaluate the amount of evolved diversity in a population.

This interaction can be split into responsiveness and inconsistency, $V_{GE} = R + I$ (Barrett *et al.*, 2005; Hall & Colegrave, 2006; Venail *et al.*, 2008). Responsiveness, R, indicates differences in environmental variances and therefore measures diversity of resource exploitation strategies (specialists vs generalists):

$$R = \sum \frac{(\sigma_{Ej} - \sigma_{Ei})^2}{2G(G-1)}$$

where G is the number of genotypes tested and σ_{Ej} and σ_{Ei} are the standard deviations of environmental responses of each clone tested across all substrates. A high responsiveness value would mean some clones are generalists and some clones are specialists that use a subset of the resources used by the generalists.

```r
# create dataframe for standard deviation per clone across
# environments
d_sd <- group_by(d_stack, clone) %>% summarise(., sd_E = sd(OD)) %>%
    data.frame(stringsAsFactors = FALSE)

# create 2 copies of this for merging later
sd_j_clone <- dplyr::rename(d_sd, clone_j = clone, sd_j = sd_E)
sd_i_clone <- rename(d_sd, clone_i = clone, sd_i = sd_E)

# create every pairwise combination of 1:n (clones/genotypes)
# for each population
d_R <- data.frame(expand.grid(clone_j = unique(d_stack$clone),
    clone_i = unique(d_stack$clone), stringsAsFactors = FALSE)) %>%
    ungroup() %>% filter(., clone_j > clone_i) %>% merge(., sd_j_clone,
    by = c("clone_j")) %>% merge(., sd_i_clone, by = c("clone_i")) %>%
    mutate(num_clones = 10)

# calculate R for each pairwise combination
d_R <- mutate(d_R, R_comb = (sd_j - sd_i)^2/(2 * num_clones) *
    (num_clones - 1)) %>% ungroup()

# sum of all the pairwise combinations
R_pop <- sum(d_R$R_comb)
R_pop
#> [1] 0.0308595
```

Inconsistency indicates non-correlations between genotypes over different environments and indicated resource specialisation.

$$R = \sum \frac{\sigma_{Ej}\sigma_{Ei}(1-\rho_{E_jE_i})}{G(G-1)}$$

where $\rho_{E_j E_i}$ is the Pearson's correlation across substrates between each pair of clones. High inconsistency means negative correlations between clones across environments (i.e. one clone will be better on substrate A than B, and vice versa for another clone).

```
# prep data for calculating correlations
d_pearson <- d_stack %>% pairwise_cor(clone, C_source, OD, upper = FALSE) %>%
    rename(clone_i = item1, clone_j = item2)

# merge dataframe to responsiveness dataframe
d_inconsist <- merge(d_pearson, sd_i_clone, by = c("clone_i"),
    all.x = TRUE) %>% merge(., sd_j_clone, by = c("clone_j"),
    all.x = TRUE) %>% mutate(., num_clones = 10, i = (sd_j *
    sd_i * (1 - correlation))/(num_clones * (num_clones - 1))) %>%
    data.frame()

I_pop = mean(d_inconsist$i)
I_pop
#> [1] 7.167263e-05
```

High inconsistency means negative correlations between clones across environments (i.e. one clone will be better on substrate A than B, and vice versa for another clone). In instances of high inconsistency and high responsiveness, clones take advantage of different resources, and some clones are specialists, and some are generalists (see Figure below).

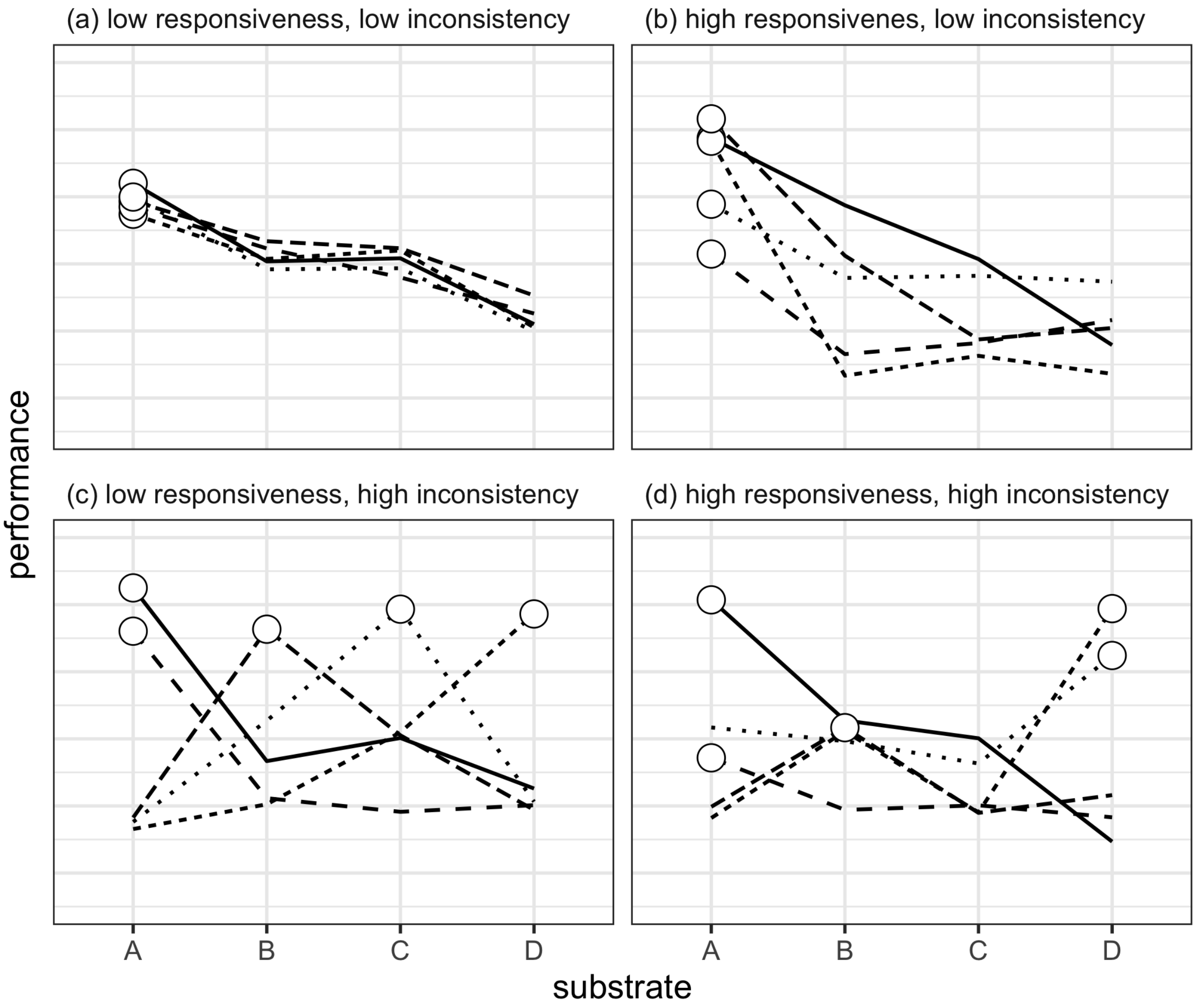

Figure 2. Scenarios of different amounts of responsiveness and inconsistency. Each dashed line represents the performance of an individual clone over substrates A to D. Dots represent the substrate on which each clone performs best on. In (a) the clones all have similar performance across all substrates and there is no C x E interaction. In (b) there is high responsiveness, but low inconsistency. The clones all perform best on resource A, but some are specialists and some generalists (they have different environmental variances). In (c) there is low responsiveness, but high inconsistency. Different clones take advantage of different resources, but there is no difference in resource exploitation strategies (they have similar environmental variances). In (d) there is high responsiveness and high inconsistency. Different clones take advantage of different resources and some clones are specialists and some are generalists.

Summary This walk-through demonstrates how all components of variance can be calculated in a reproducible way for a single population. These can easily be scaled up to calculating them for multiple populations and treatments using **group_by()** and **summarise** in the **tidyverse** packages.

References

- Barrett RDH, MacLean RC, Bell G. 2005 Experimental evolution of Pseudomonas fluorescens in simple and complex environments. Am. Nat. 166, 470–480.
- Hall AR, Colegrave N. 2007 How does resource supply affect evolutionary diversification? Proc. Biol. Sci. 274, 73–78.
- Venail PA, MacLean RC, Bouvier T, Brockhurst MA, Hochberg ME, Mouquet N. 2008 Diversity and productivity peak at intermediate dispersal rate in evolving metacommunities. Nature 452, 210-214.
